# Using the derived 28-joint disease activity score patient-reported components (DAS28-P) index as a discriminatory measure of response to disease-modifying anti-rheumatic drug therapy in early rheumatoid arthritis

**DOI:** 10.1186/s41927-022-00299-3

**Published:** 2022-11-15

**Authors:** Huai Leng Pisaniello, Samuel L. Whittle, Susan Lester, Fiona Menz, Robert Metcalf, Leah McWilliams, Catherine L. Hill, Susanna Proudman

**Affiliations:** 1 Discipline of Medicine, Faculty of Health and Medical Sciences, The University of Adelaide, Adelaide, SA Australia; 2 Rheumatology Research Group, Basil Hetzel Institute for Translational Health Research, The Queen Elizabeth Hospital, Woodville South, SA Australia; 3 Rheumatology Unit, The Queen Elizabeth Hospital, Woodville South, SA Australia; 4 Flinders Medical Centre, Bedford Park, SA Australia; 5 Rheumatology Unit, Royal Adelaide Hospital, Adelaide, SA Australia

**Keywords:** DAS28-P index, Rheumatoid arthritis, Pain, Patient global assessment

## Abstract

**Background:**

The 28-joint disease activity score (DAS28) is a widely used measure to assess disease activity in rheumatoid arthritis (RA). The DAS28-P index, a derived proportion of the patient-reported components (joint tenderness and patient global assessment) within the DAS28, has been utilized as a discriminatory measure of non-inflammatory pain mechanisms in RA. This study aimed to evaluate the use of the DAS28-P index as a predictor of treatment response in early RA.

**Methods:**

Patients with early RA enrolled in a supplemental fish oil clinical trial received a combination of disease-modifying anti-rheumatic drugs (DMARDs) according to a ‘treat-to-target’ protocol. First, consecutive measures of the DAS28-P index, derived from the DAS28-erythrocyte sedimentation rate (DAS28-ESR), at each visit over a 1-year period were estimated for each patient. Then, distinct subgroups of treatment responders based on the trajectories of the DAS28-P indices were identified using bivariate k-means cluster analysis. Data on baseline predictors as well as longitudinal outcomes of disease impact and DMARD use over a 1-year period and radiographic progression over a 3-year period were collected and analyzed using a random intercept, population-averaged generalized estimating equation model.

**Results:**

121 patients were included (74% female; mean age of 57; median of 16 weeks of active disease) and a 3-cluster model was identified—the ‘Responders’ group (n = 58; 48%), the ‘Partial Responders’ group (n = 32; 26%), and the ‘Non-Responders’ group (n = 31; 26%). The ‘Partial Responders’ group had consistently higher proportions of the DAS28-P index throughout the study period and had minimal radiographic progression over time, with the lowest joint erosion score of 0.9 [95% confidence interval (CI) 0.2, 1.6], observed at the 3-year follow-up. At 52 weeks, the methotrexate dose was higher for both ‘Partial Responders’ and ‘Non-Responders’ groups (18.5 mg [95% CI 15.5, 21.5] and 18.6 mg [95% CI 15.3, 21.8] respectively), when compared with the ‘Responders’ group (12.8 mg [95% CI 14.7, 20.9]).

**Conclusions:**

Persistently high DAS28-P index scores are useful to distinguish poor patient global assessment and excessive treatment escalation in early RA, suggestive of underlying non-inflammatory pain contributing to higher disease activity score. Early identification of patients with discordant subjective and objective components of composite disease activity measures may allow better tailoring of treatment in RA.

## Background

Rheumatoid arthritis (RA) is a chronic autoimmune inflammatory disease that results in joint pain and swelling, as well as other peri-articular and extra-articular systemic manifestations [1]. RA characteristically affects the small joints of the hands (the metacarpophalangeal joints and the proximal interphalangeal joints), wrists, knees and feet [1]. In recent years, the long-term outcomes for RA have improved significantly, largely driven by advances in disease-modifying anti-rheumatic drugs (DMARDs), in particular biologic therapy, and with the implementation of ‘early diagnosis’ and ‘treat-to-target’ (T2T) approaches [1–4]. Pain remains a cardinal feature in RA and historically, pain mechanisms in RA have been attributed solely to activation of the peripheral nociceptive pathways by the underlying joint inflammation [5]. Consequently, the notions of controlling the disease activity with DMARDs and achieving disease remission have always been the cornerstone of pain management in RA, although this approach does not hold true for some patients with persistent pain.

In fact, the pain mechanism in RA involves a complex interaction between the inflammatory process in the joints and a combination of both the activation of the peripheral nociceptors and the peripheral and central modulation of nociceptive and other inputs [5]. The presence of joint pain despite apparent good control of synovial inflammation implies that mechanisms other than pure nociception are important in the overall pain experience in RA. The relative contributions of different peripheral and central mechanisms may vary between individuals with RA. For instance, a Swedish population-based cohort study found that nearly one-third of patients with early RA had persistent pain despite effective control of the joint inflammation. This finding was strongly predicted by having both functional impairment and low C-reactive protein (CRP) level at baseline [6]. Additionally, over-estimation of disease activity scores by non-inflammatory pain has been observed as a common occurrence in RA, irrespective of the timing of initiating or escalating treatment [7]. Identifying non-nociceptive pain during the treatment course for RA is crucial as overtreatment in patients with persistent pain unrelated to the underlying inflammation is unfavorable and can be harmful and is especially likely to occur in the context of a T2T approach.

In clinical practice and clinical trials, particularly in the modern T2T approach, the monitoring of disease activity and treatment response in RA is commonly performed by using the disease activity score 28-joints (DAS28) [8, 9]. DAS28 is a composite score derived collectively from the objective measures as assessed by the clinician (i.e., swollen joint counts (SJCs) and the acute-phase response (erythrocyte sedimentation rate (ESR) or CRP level) and the patient-reported measures (i.e., tender joint counts (TJCs) and global health as assessed by using a visual analogue scale (VAS) of patient-reported disease activity (VAS-GH)) [10, 11]. These patient-reported measures within the DAS28 composite score, representative of the patient global assessment (PGA), are more susceptible to individual-level variation due to factors other than inflammation alone. When it comes to interpreting the DAS28 score, careful interpretation of the PGA is important, especially in patients with pain driven predominantly by centrally augmented pain mechanisms [12, 13]. Various composite disease activity measures are used in the T2T paradigm. DAS28 remains in common clinical use although other measures such as the Simplified Disease Activity Index (SDAI) and the Clinical Disease Activity Index (CDAI) have been the preferred composite disease activity measures in recent years [14–16]. Non-inflammatory pain experienced by patients in RA may not be well captured by the overall scoring of the current composite disease activity measures, and the decomposition of a composite measure into subjective and objective components may be relevant to other measures, such as the SDAI, although this remains an open research question. McWilliams and colleague proposed that the use of the DAS28-P index, defined as “a derived measure of the proportion for the contribution of the patient-reported outcomes (TJC and VAS-GH) to the total DAS28 score”, is considered a useful discriminatory index of non-inflammatory pain mechanisms in RA [12, 17]. A higher DAS28-P index was shown to predict less pain improvement in an early RA cohort at 12 months, when adjusted for baseline pain scores [12]. Similarly, a higher DAS28-P index was correlated with widespread pressure-induced pain sensitivity in established RA and with the fibromyalgia survey score [18].

In this study, building on existing research utilizing the DAS28-P index, we hypothesized that in an early RA cohort with DMARDs initiated and modified to meet a pre-defined level of disease activity, the DAS28-P index is useful in discriminating non-inflammatory pain mechanisms in early RA. We first aimed to identify different disease trajectories for each participant in this early RA cohort by using the objective and subjective components of the DAS28-ESR. Next, we aimed to assess the impact of using the subjective components of the DAS28-ESR, and therefore, the role of the DAS28-P index in monitoring disease activity and in determining the trajectory of DMARD use in a T2T approach.

## Methods

### Participants

Our study included a subset of participants recruited for a randomized controlled trial (RCT) of fish oil use in early RA. DAS28 was the most widely used composite disease activity measure in RA during the conduct of the study. As described in the original study, consecutive patients aged 18 years and older with early onset RA (defined as symptomatic polyarthritis of less than 12 months, SJC ≥ 3, TJC ≥ 6, ESR > 28 mm/h, and/or CRP > 10 mg/dL) diagnosed at the Rheumatology Clinic, Royal Adelaide Hospital (RAH), South Australia were recruited. These DMARD-naïve patients, who fulfilled the diagnosis of RA according to the 1987 revised American College of Rheumatology (ACR) criteria, were enrolled, and screened for eligibility to enter a double-blind RCT of high dose fish oil versus low dose fish oil. The exclusion criteria included DMARD use other than anti-malarials, use of anti-malarials for more than one month, recent seroconversion to parvovirus, Ross River, Barmah Forest, or rubella viruses, history of positive anti-nuclear antibody with a titre of ≥ 1:320, history of positive hepatitis B, hepatitis C or human immunodeficiency virus (HIV), known sensitivity to methotrexate, sulfasalazine or hydroxychloroquine, and history of systemic disease likely to increase risk of toxicity to 1 or more of these DMARDs. The study was approved by the RAH Research Ethics Committee (Research Protocol No: 981105).

### Study protocol

The full details of the original study cohort, study design, study treatment strategy and results have been previously published elsewhere [19–21]. Alongside the randomization of receiving high dose vs low dose fish oil in the study, patients commenced DMARDs with dose adjustments based on a T2T treatment approach. In brief, triple DMARD therapy comprised methotrexate 10 mg orally weekly, folic acid 500mcg daily, sulfasalazine 500 mg daily, with dose increment over 4 weeks to 1 g twice daily and hydroxychloroquine 200 mg twice daily. The methotrexate dose was up-titrated to a maximum dose of 25 mg weekly administered subcutaneously to achieve disease remission based on pre-specified disease activity criteria [19, 20]. The addition of leflunomide was considered when maximal tolerated doses of triple DMARD therapy were achieved. Use of oral glucocorticoids and non-steroidal anti-inflammatory drugs (NSAIDs) was actively discouraged during the study, and if commenced at study inception, doses were gradually tapered and ceased where possible. Parenteral glucocorticoid use during the study was allowed as clinically indicated.

### Data collections and measurements

Patients were reviewed every 3–6 weeks and measures of disease activity were taken at each follow-up visit. Specific to the aims of our study, we obtained data pertaining to longitudinal measures of disease activity and disease impact for the first 52 weeks. Both objective components (SJCs (28 joints) and ESR) and subjective components (TJCs (28 joints) and VAS-GH) of the DAS28-ESR captured for all visits (baseline and follow-up) for the first 52 weeks were reviewed and analyzed separately. On average, there were 9.7 visits per patient. A 100-mm VAS was used to assess each of these domains—global health (VAS-GH), pain and fatigue. DAS28-P index, defined as the fraction of the total DAS28-ESR contributed by the subjective components of the DAS28-ESR, was calculated for each visit. The modified Health Assessment Questionnaire (mHAQ) and 36-Item Short Form Survey (SF-36) were used at each visit to assess function and yearly to assess quality of life, respectively. A validated 5-item Rheumatology Attitudes Index (VALI-RAI) helplessness subscales (5–30) scoring system was used at each visit to evaluate patients’ views of helplessness in coping with their arthritis [22]. X-rays of the hands and feet were performed for each patient annually for 3 years. In a blinded, chronological fashion, for each time point out to 3 years, two independent observers assessed the presence of joint erosion in these radiographs of hands and feet using the modified Sharp/van der Heijde (SHS) method [21, 23].

### Statistical analysis

For the descriptive data, the categorical variables were summarized as absolute numbers and proportions in percentages, whereas the continuous variables were summarized as means with standard deviation or median with interquartile range. For the overall pairwise comparisons between the identified clusters, p-values for normally distributed continuous variables were calculated using one-way ANOVA and p-values for non-normally distributed continuous variables were analyzed using Kruskal–Wallis one-way ANOVA. Categorical variables were analyzed using Pearson’s chi square and 2-sided Fisher’s exact tests for concomitant rheumatological diseases, such as fibromyalgia.

To identify subgroups of patients with different profiles of DAS28 trajectories, a bivariate longitudinal k-means clustering analysis was performed using both the individual objective and subjective DAS28-ESR component scores. This non-parametric clustering analysis was performed using the R package, klm3d [24]. Three treatment responder subgroups were selected according to our prior hypothesis that RA patients with persistent pain would be differentiated from both good- and non-responders.

For the identified cluster subgroups, the longitudinal outcome measures were analyzed by using a random intercept, population-averaged generalized estimating equation (GEE) model (a longitudinal generalized linear model), with an exchangeable correlation matrix and robust standard errors. In this GEE model, different regression analyses were applied for different outcome measures. For instance, binomial regression was used for both leflunomide and NSAIDs use and the presence of depression, Poisson regression for methotrexate use and negative binomial regression was used for the total joint erosion scores. The remaining outcome measures were analyzed by Gaussian regression, except the DAS28-P index scores (range of 0–1), which were analyzed by a probit fractional regression, with standard errors clustered by each patient. The outcomes for the total joint erosion scores were measured at multiple, irregularly spaced visits over the 12 months of follow-up, with an average of 9.8 months per patient. Therefore, restricted, orthogonal cubic splines (with 3 degrees of freedom (d.f.) for the main effect, and an additional 2 d.f. for interaction effects) were applied to model the responses over time. As a result, the differences between the subgroups over time were assessed by joint Wald tests of the appropriate regression coefficients.

For the SF-36 data, the scores for each nine domains were converted to Physical Component Scores and Mental Component Scores using the Stata sf36.ado module [25]. To allow for between-domain comparisons of results, each SF-36 domain scale (0–100) was transformed to a norm-based scale with a mean of 50 and standard deviation of 10, using direct age- and gender-standardization to the South Australian (SA) population norms from the 1995 National Health Survey [26].

All results were interpreted as predicted marginal means (i.e., on the original response scale) with linear contrasts used to assess differences between response from each subgroup at specific time points. The clustering analysis was performed using R version 3.2.0 and the remaining statistical analyses were performed using Stata v16.1 (StataCorp LLC, TS, USA).

## Results

A total of 121 patients were included in the final analysis for this study, with 1220 observations captured from baseline to 52 weeks. These patients were predominantly female (74%) with a mean age of 57 years and had a median of 16 weeks of symptomatic polyarthritis at baseline and 54% were seropositive for anti-cyclic citrullinated peptide antibodies (ACPAs). Among these 121 patients, the k-means clustering analysis generated 3 subgroups of patients according to the 52-week trajectories of these 3 outcome measures: the overall DAS28-ESR, the objective components of the DAS28 and the subjective components of the DAS28. These subgroups were classified as Group 1—‘Responders’, Group 2—‘Partial Responders’ and Group 3—‘Non-Responders’. The baseline characteristics for each of these groups are outlined in Table 1.

**Table 1:** Baseline characteristics of participants included in the study

Descriptor	Group 1	Group 2	Group 3	All
Classification	Responders	Partial responders	Non-responders	
N (%)	58 (48%)	32 (26%)	31 (26%)	121
Age: mean (SD)	56 (16)	54 (15)	63 (11)	57 (15)
Females (%)	45 (78%)	25 (78%)	19 (61%)	89 (74%)
BMI: mean (SD)	26.4 (5.1)	27.4 (4.8)	30.6 (7.6)*	27.7 (6.0)
ACPA positive (%)	31/55 (56%)	14/31 (45%)	18/31 (58%)	63/117 (54%)
RF positive (%)	39/55 (71%)	13/31 (42%)	18/31(58%)	70/117 (60%)
Smoking (%)				
Never	29 (50%)	16 (50%)	7 (23%)	52 (43%)
Former	20 (34%)	14 (44%)	15 (48%)	49 (41%)
Current	9 (16%)	2 (6%)	9 (29%)	20 (17%)
Odds ratio_ordinal_ (95% CI) for the likelihood of smoking	*1.2 (0.5, 2.7)*	*1*	3.6 (1.4, 9.2)*	
Weeks polyarthritis: median (IQR)	16 (12, 24)	16 (12, 24)	20 (12, 28)*	16 (12, 24)
Randomized to fish oil (%)	38 (66%)	20 (63%)	17 (54%)	75 (62%)

The odds ratio (highlighted in italics) was calculated for the likelihood of smoking

^*^Significantly different from Group 2 (p < 0.05)

*SD* standard deviation, *BMI* body mass index, *ACPA* anti-cyclic citrullinated peptide antibody, *RF* rheumatoid factor, *CI* confidence interval, *IQR* interquartile range

The predicted marginal means and their corresponding 95% confidence intervals (CI) for all outcome measures of disease activity and disease impact at baseline and at week 52 for each responder group are summarized in Table 2. Overall, at baseline, the participants in the ‘Non-Responders’ group were older, had a higher BMI and were more likely to have smoked (Table 1). The ‘Responders’ group had the lowest baseline DAS28-ESR and the ‘Partial Responders’ group had the highest baseline DAS28-P index (Table 2).

**Table 2:** Predicted marginal means (95% confidence intervals, CI), by responder group, for all outcome measures at baseline and at week 52

	Baseline			52 Weeks		
	Group 1	Group 2	Group 3	Group 1	Group 2	Group 3
	Responders	Partial responders	Non-responders	Responders	Partial responders	Non-responders
DAS28-ESR	5.1 (4.9, 5.4)*	6.1 (5.7, 6.5)	6.3 (5.9, 6.6)	2.3 (2.1, 2.6)*	3.9 (3.3, 4.4)	5.1 (4.7, 5.5)*
DAS28-ESR objective component	3.0 (2.9, 3.2)	3.0 (2.7, 3.2)	3.4 (3.2, 3.6)*	1.7 (1.5, 1.8)	1.7 (1.5, 1.9)	2.8 (2.7, 3.0)*
DAS28-ESR subjective component	2.1 (1.9, 2.3)*	3.1 (2.9, 3.4)	2.9 (2.7, 3.1)	0.6 (0.5, 0.8)*	2.1 (1.8, 2.5)	2.3 (2.0, 2.6)
DAS28-P	0.40 (0.38, 0.43)*	0.52 (0.50, 0.55)	0.45 (0.43, 0.48)*	0.23 (0.19, 0.28)*	0.56 (0.52, 0.59)	0.43 (0.39, 0.47)*
mHAQ	0.61 (0.49, 0.73)*	0.91 (0.75, 1.07)	0.82 (0.64, 0.99)	0.11 (0.04, 0.18)*	0.47 (0.25, 0.67)	0.48 (0.32, 0.63)
Fatigue	36.1(30.6, 41.6)*	66.7 (58.7, 74.6)	53.7 (45.1, 62.2)*	18.9 (12.7, 25.1)*	44.6 (35.0, 54.1)	48.3 (39.4, 57.1)
Helplessness	13.0 (11.8, 14.3)*	16.1 (14.2, 17.9)	15.4 (13.7, 17.1)	8.6 (7.6, 9.6)*	13.0 (11.4, 14.6)	13.0 (11.4, 14.7)
SF-36						
Physical component score	37.5 (35.1, 39.9)*	32.1 (29.9, 34.3)	33.5 (30.6, 36.4)	47.6 (45.3, 50.0)*	38.5 (35.4, 41.6)	35.7 (32.9, 38.6)
Mental component score	43.5 (40.8, 46.3)*	35.3 (33.3, 38.3)	37.0 (32.8, 41.2)	48.4 (40.8, 46.3)*	41.4 (37.6, 45.1)	40.1 (35.9, 44.4)
Methotrexate dose				12.8 (14.7, 20.9)*	18.5 (15.5, 21.5)	18.6 (15.3, 21.8)
Leflunomide use (%)				4% (0, 10)*	16% (2, 30)	40% (21, 59)
Cumulative glucocorticoid dose, mg: median (IQR)				171 (100, 250)	199 (150, 450)	297 (211, 484)
Total erosion score	0.5 (0.3, 0.8)	0.5 (0.1, 1.0)	1.0 (0.3, 1.7)	1.2 (0.6, 1.7)	0.9 (0.2, 1.7)	2.4 (1.1, 3.6)*

The cumulative glucocorticoid dose was calculated in milligrams(mg) of prednisolone equivalent

*DAS28-ESR* disease activity score 28-joints-Erythrocyte Sedimentation rate, *mHAQ* modified Health Assessment Questionnaire, *SF-36* 36-item short form survey, *IQR* interquartile range

^*^Significantly different from Group 2 (p < 0.05)

**Fig. 1:**
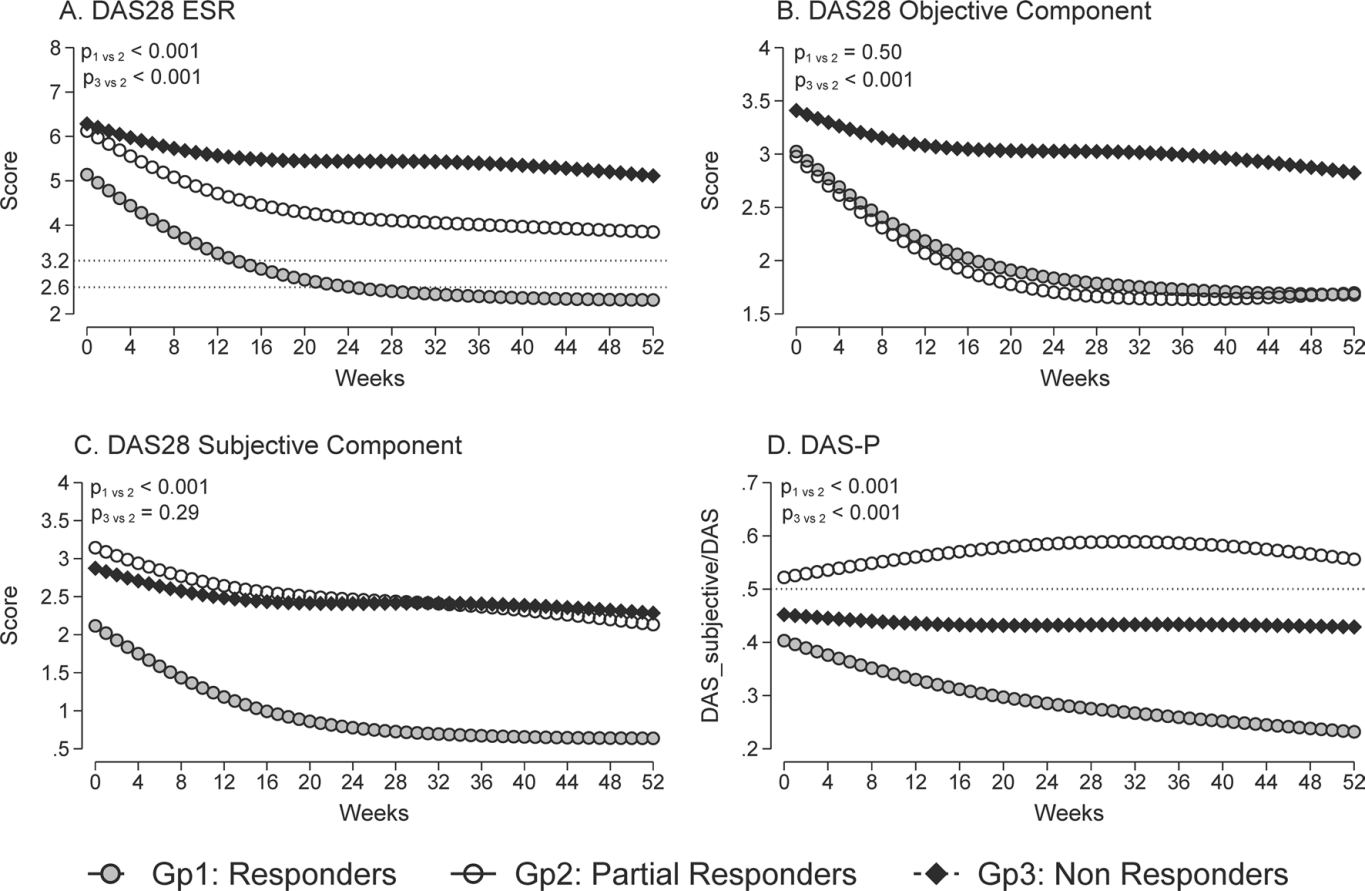
DAS28 and DAS28 component scores (predicted marginal means) during the first year of treatment for the three responder groups. **A** DAS28-ESR **B** DAS28—Objective Component **C** DAS28—Subjective Component **D** DAS28-P (an index defined by DAS28—Subjective Component/DAS28-ESR)

For the overall disease activity trajectories during the first year of treatment, the baseline mean DAS28-ESR for the whole cohort was 5.7 (s.d. 1.2). At 52 weeks, the DAS28-ESR score in the ‘Partial Responders’ group was 3.9 [95% CI 3.3, 4.4], which was worse than the ‘Responders’ group (2.3 [95% CI 2.1, 2.6; p < 0.05]) and better than the ‘Non-Responders’ group (5.1 [95% CI 4.7, 5.5; p < 0.05]). These results were largely due to the lower subjective DAS28 score of 0.6 [95% CI 0.5, 0.8; p < 0.05] for the ‘Responders’ group and higher objective DAS28 score of 2.8 [95% CI 2.7, 3.0; p < 0.05] for the ‘Non-Responders’ group. As shown in Fig. 1, the overall DAS28-P mean scores during the first year of treatment were consistently above 0.5 for the ‘Partial Responder’ group in comparison with the ‘Responders’ and ‘Non-Responders’ groups, with the subjective DAS28 score being the major contributor to the total DAS28 score. Notably, the trajectory of the objective DAS28 scores in the ‘Partial Responder’ group was similar to the ‘Responder’ group (Fig. 1B), whereas the trajectory of the subjective DAS28 scores in the ‘Partial Responder’ group was similar to the ‘Non-Responder’ group (Fig. 1C).

**Fig. 2:**
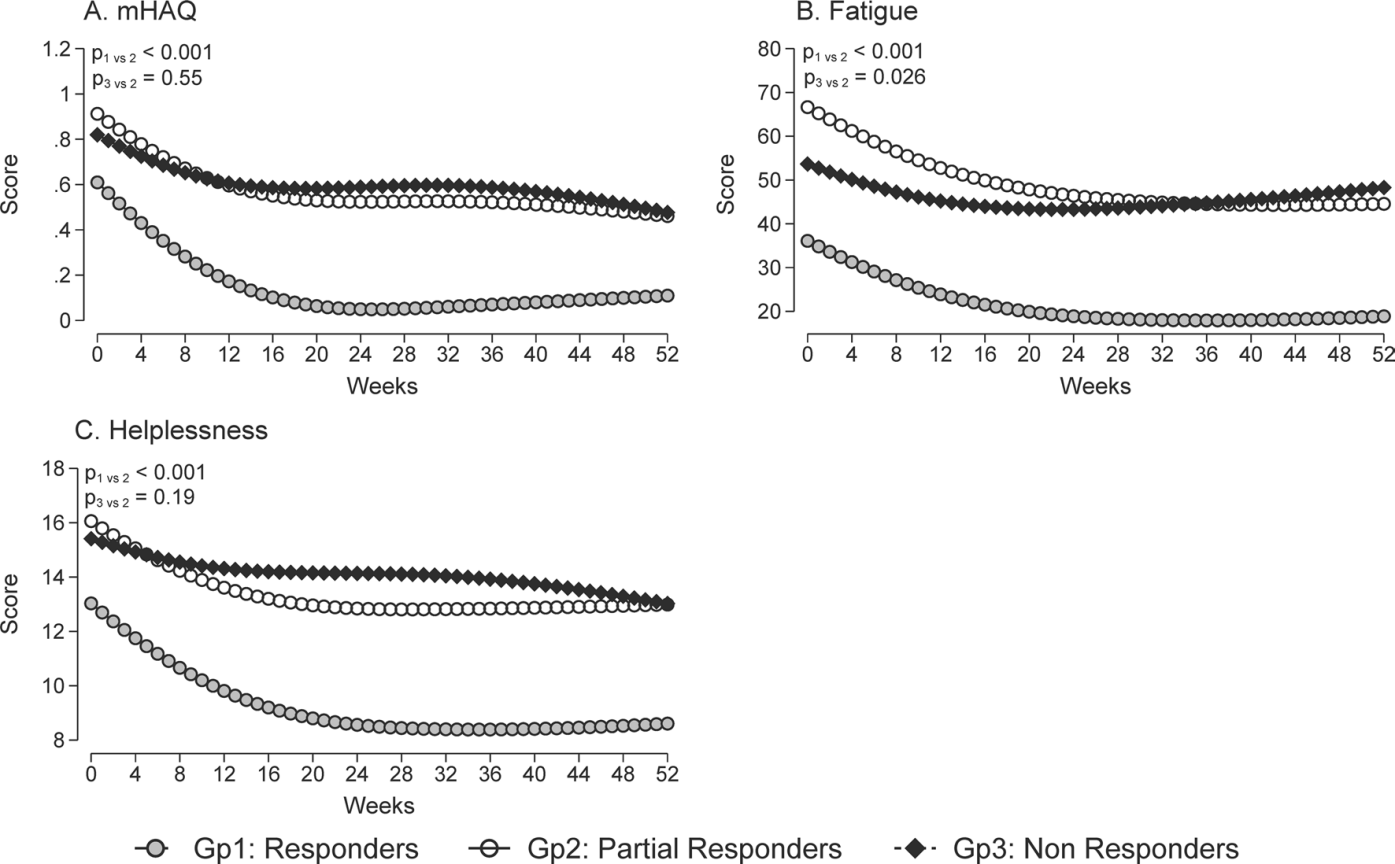
Self-reported scores (predicted marginal means) during the first year of treatment for the three responder groups: **A** modified Health Assessment Questionnaire (mHAQ) disability **B** Fatigue (measured in 0–100 mm visual analogue scale) **C** Helplessness in coping with arthritis (measured in a 5–30 subscale scoring system in a Validated 5-item Rheumatology Attitudes Index (VALI-RAI))

In terms of self-reported outcome measures of disease impact during the first year of treatment, the overall mean mHAQ at baseline for the whole cohort was 0.75 (s.d. 0.54). As shown in Fig. 2, the ‘Responders’ group had consistently lower mHAQ scores when compared to the ‘Partial Responders’ group (p < 0.001). Similarly, the ‘Responders’ group had consistently lower levels of fatigue and helplessness scores (p < 0.001 for both measures) when compared to the ‘Partial Responders’ group. Both the physical and mental component scores of the SF-36 were statistically better in the ‘Responders’ group (means of 47.6 [95% CI 45.3, 50.0] and 48.4 [95% CI 40.8, 46.3] respectively).

**Fig. 3:**
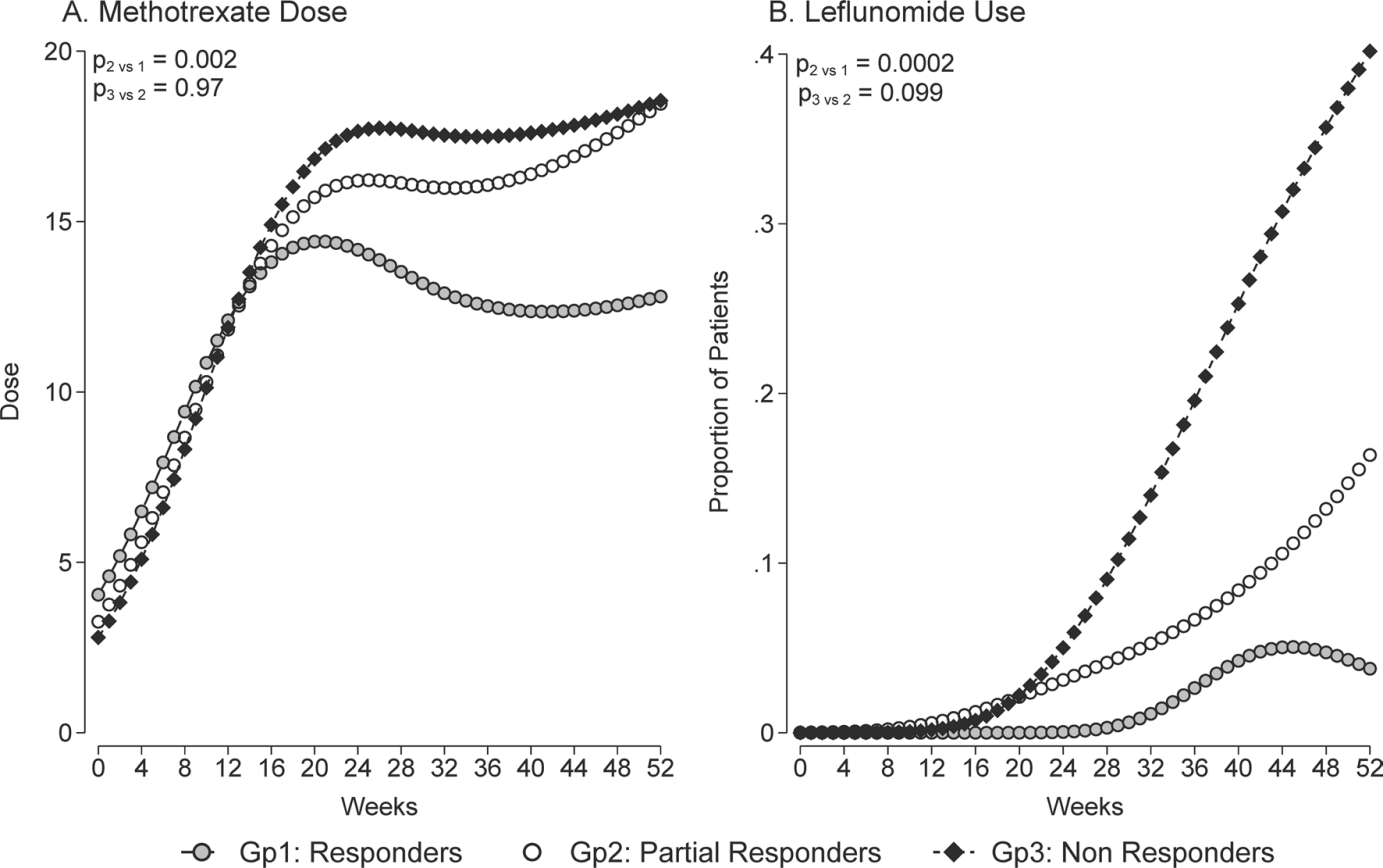
DMARD treatment (predicted marginal means) during the first year for the three responder groups: **A** Methotrexate (MTX) Dose (in milligram, mg of weekly dosing) **B** Leflunomide Use (proportion of patients)

In terms of DMARD treatment comparisons between the responder groups during the first year of treatment, based on the T2T approach, the methotrexate dose profile was higher in the ‘Partial Responders’ group compared to the ‘Responders’ group, but was comparable to the ‘Non-Responders’ group (Fig. 3A). At 52 weeks, the methotrexate dose (in milligram, mg) was 12.8 mg [95% CI 14.7, 20.9] for the ‘Responders’ group, 18.5 mg [95% CI 15.5, 21.5] for the ‘Partial Responders’ group and 18.6 mg [95% CI 15.3, 21.8] for the ‘Non-Responders’ group. The leflunomide use profile in the ‘Partial Responders’ group was intermediate between the ‘Responders’ and ‘Non-Responders’ groups (Fig. 3B). Although NSAID use was permitted during the study period, there were no significant differences in terms of NSAID use profile between the three responder groups. In patients who were given glucocorticoid during the study period, the cumulative glucocorticoid doses (expressed in milligrams of prednisolone equivalent) at week 52 were relatively low among the three responder groups (p = 0.022, Kruskal–Wallis rank test for equality of populations). The median cumulative glucocorticoid dose was the highest in the ‘Non-Responders’ group (297 mg, IQR 211, 284; n = 24), compared to the ‘Partial Responders’ group (199 mg, IQR 150, 450; n = 24) and the ‘Responders’ group (171 mg, IQR 100, 250; n = 27) (Table 2).

**Fig. 4:**
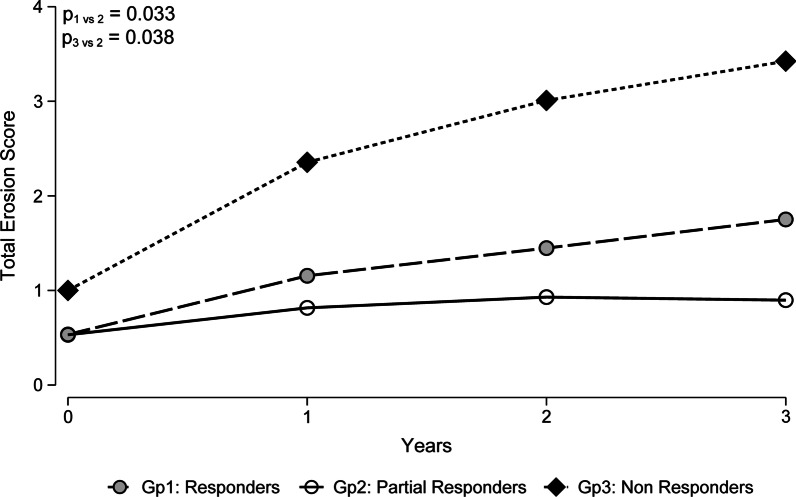
Total Erosion Scores (predicted marginal means) over 3 years of follow-up for the three responder groups

The total joint erosion scores at baseline and 12 months follow-up are outlined in Table 2. While the comparison of total joint erosion scores between the ‘Responders’ and ‘Partial Responders’ groups over all time points was significant (p = 0.033), there were no significant differences at any individual time point (Fig. 4). However, the comparison between the ‘Partial Responders’ and ‘Non-Responders’ groups was significant (p < 0.05) at each follow-up time point (years 1, 2 and 3). At year 3, the total joint erosion score in the ‘Responders’ group was 1.8 (95% CI 0.8, 2.7), compared to 0.9 (95% CI 0.2, 1.6) in the ‘Partial Responders’ group and 3.4 (95% CI 1.7, 5.2) in the ‘Non-Responders’ group.

## Discussion

Composite disease activity measures such as the DAS28 are routinely used in rheumatology practice to monitor the disease trajectory in RA. In this study of an early RA cohort managed with a T2T approach, we identified three distinct subgroups of patients with different disease trajectories over 12 months by clustering each component of the DAS28 (the overall score, and both the objective and subjective components of the score). Of the 121 study participants, at 52 weeks, nearly half of them were in disease remission (the ‘Responders’ group), and the other half of the study cohort continued to have moderate-to-high disease activity, with 26% in the ‘Partial Responders’ group and 26% in the ‘Non-Responders’ group. When we examined the components of the DAS28, both ‘Responders’ and ‘Partial Responders’ groups had similar DAS28 objective component mean scores at baseline and at 52 weeks, and yet, these two subgroups had different disease trajectories. In fact, the relatively high total mean DAS28-ESR scores for the ‘Partial Responders’ group at baseline and at 52 weeks were largely driven by the reporting of high DAS28 subjective component scores, as highlighted by the consistently higher proportions of the DAS28-P index throughout the study period when compared to both ‘Responders’ and ‘Non-Responders’ groups. Despite receiving similar T2T therapy, these findings in the ‘Partial Responders’ group reflect ongoing patient-reported concerns about their disease trajectories disproportionate to the underlying disease inflammation.

In our study, both ‘Partial Responders’ and ‘Non-Responders’ groups reported similar worsening of disease impact throughout the study period, when compared to the ‘Responders’ group, as demonstrated in the mHAQ scores, the level of fatigue and helplessness scores. Apart from the higher level of fatigue in the ‘Partial Responders’ group, both ‘Partial Responders’ and ‘Non-Responders’ groups were indistinguishable at baseline, even in the DAS28 subjective component scores. Evidently, these two subgroups differed in the trajectories of the DAS28 objective component scores and the DAS28-P proportion indices. Again, according to the DAS28-P index, these findings suggest a predominance of non-inflammatory pain mechanisms in the ‘Partial Responders’ group and failure of treatment and ongoing active disease in the ‘Non-Responders’ group at baseline and throughout the study. Although a difference in the DAS28-P was seen between the ‘Partial Responders’ group and the ‘Non-Responders’ group (Fig. 1D), the difference between each group may not be sufficient to reliably categorize individual patients at any time point. Other concomitant chronic pain conditions, such as osteoarthritis and fibromyalgia, could be confounders for persistent pain in this study cohort, and although beyond the scope of our study, their contribution to non-inflammatory pain in early RA would be relevant in any future analysis. Although the DAS28-P index can be used as a discriminatory measure of non-inflammatory pain in RA, our study highlights that baseline DAS28-P does not predict trajectory of RA disease activity in individuals, which was not previously examined in the original study proposing the use of DAS28-P index [12].

Overtreatment is a potential risk in the modern treatment era for patients diagnosed with early RA, especially in the T2T approach [27]. In this study, there was a substantial increase in both the methotrexate mean dose and the proportions of leflunomide users in both ‘Partial Responders’ and ‘Non-Responders’ groups. In detail, dose increments for both of these DMARDs were seen at week 16, a typical time period for deciding any change in dosing, and subsequently the doses were gradually up-titrated to the maximum recommended target doses, as dictated by the serial DAS28 scores. Similarly, despite the analysis of only a subset of the study cohort, the cumulative dose of glucocorticoid use in the ‘Non-Responders’ group was substantively higher compared to the other two subgroups. Consequently, these subgroups with disproportionate dose titration and disease activity could be at risk of DMARD-related toxicity in the intermediate- and long-term. Likewise, a recent study by Wallace and colleague revealed two thirds of established rheumatoid arthritis patients had persistent glucocorticoid use, especially in those with high fibromyalgianess [28]. In our study, we observed that a higher DAS28-P in both ‘Partial Responders’ and ‘Non-Responders’ groups was associated with higher exposure to combination DMARD therapy. Relying on the use of only the composite DAS28 score might lead to overtreatment, which could be mitigated by understanding the relative contributions of subjective and objective measures to the total composite score. In addition, escalation to biologic DMARDs in these subgroups may occur, which may result in higher societal and health care cost and unnecessary immunosuppression. Future studies examining the use of conventional synthetic DMARDs beyond a 1-year period and the timing of switching to biologic DMARD in these subgroups of early RA cohort may help to further characterize the impact of the T2T treatment approach in those with persistent non-inflammatory pain in RA.

Furthermore, in our study, despite not achieving the DAS28 indicative of low disease activity/disease remission, the ‘Partial Responders’ group had the lowest joint erosion scores serially over 3 years, demonstrating no progression of erosive disease. This is consistent with their low levels of disease inflammation following treatment, as reflected by the overall DAS28 objective component score. This finding underscores the risk of unnecessary overtreatment in a ‘partially-responsive’ subgroup of patients with early RA, in whom additional immunosuppressive agents will not alleviate non-inflammatory symptoms. Adjuvant interventions that target non-inflammatory pain rather than relying on immunosuppressive therapies are likely warranted in this group of patients with suboptimal disease control despite no objective evidence of ongoing inflammation [29, 30]. With regards to radiographic progression, the ‘Responders’ group had higher erosion risks compared to the ‘Partial Responders’ group, although the ‘Responders’ group had the overall lowest subjective DAS28-ESR scores. This may reflect the recognised phenomenon of progressive structural damage even when objective measures of disease activity are low/normal, and highlights the importance of assessing radiographic outcomes in addition to both subjective and objective disease activity measures in RA [31–33]. Judicious interpretation of all these outcome measures may lower the risk of overtreatment in those with high DAS28-P and, conversely, undertreatment in those with low DAS28-P.

In the modern T2T strategies in achieving disease remission in RA, we are yet to have mutually exclusive composite measures to incorporate disease outcome measures important to both clinicians and patients. From the patient’s perspective, disease remission comprises both resolution of disease inflammation and alleviation of symptoms related to the disease. Although the PGA within the DAS28 has been considered the cornerstone of determining the patient-reported disease remission, the role of PGA remains contentious. A recent large individual patient data meta-analysis evaluating the impact of PGA in the definition of disease remission and as a predictor of radiographic damage in RA concluded that the current DAS28 remission definition that includes the PGA, is better than a definition that excludes PGA for predicting a good functional outcome but reduces the predictive accuracy for radiological outcomes, raising concerns for risk of overtreatment [34]. In a large multinational study using the METEOR database of patients on biologic DMARDs for RA, the PGA remained high in those in remission, with the danger of further unnecessary immunosuppression [35]. Ferreira and colleagues have proposed a dual T2T strategy, which comprises the management of disease inflammation (biologic remission) and the management of disease impact (symptom remission) to guide treatment in RA [36]. For biologic remission, alongside the dual T2T, the author recommended the use of 3-variable remission—SJC, TJC and CRP [37]. For symptom remission, the author suggested further validation of the PGA with the use of the Rheumatoid Arthritis Impact of Disease (RAID) score [37]. This additional patient-reported measure in early RA may provide early insight at the start of DMARD initiation, with early adjuvant interventions to be provided to those who are likely to have persistent non-inflammatory pain.

Our study has some strengths and limitations. Our study examined patients who were definitively diagnosed with early RA as they were recruited through strict RCT inclusion criteria. We were able to differentiate patients with persistent pain (the ‘Partial Responders’ group) from the ‘Non-Responders’ group, a difference that was not shown in the previous study using the DAS28-P index [12]. It included a relatively small cohort of patients with early RA. Irrespective, we had adequate study size and repeated measures to provide representative subgroups of patients with different trajectories to evaluate the utility of DAS28-P index as predictor of treatment response in the first year after diagnosis of RA. In addition, the study participants were mainly recruited from a single-center rheumatology unit, which may introduce selection bias in terms of the residency of the patients and their corresponding education levels and socio-economic status.

In summary, in this well-characterized early RA cohort managed with a T2T approach within the first year, the DAS28-P index can be used as a discriminatory measure of non-inflammatory pain in RA, but baseline DAS28-P does not necessarily predict trajectory in individuals. Concurrent assessment of both objective and subjective components of the DAS28 is likely to be most informative when it comes to tailoring of therapy in patients with RA, especially in treatment escalation. Most importantly, early identification of patients with discordant subjective and objective outcomes may facilitate optimal shared decision-making regarding DMARD and pain management. Additional clinical assessment and communication are warranted when there is a suspicion of ongoing non-inflammatory pain despite adequate control of disease inflammation.

## Data Availability

All data generated or analyzed during this study are included in this published article.

## Abbreviations

ACR: American College of Rheumatology
ACPAs: Anti-cyclic citrullinated peptide antibodies
CI: Confidence intervals
CRP: C-reactive protein
DAS28: Disease activity score 28-joints
d.f.: Degrees of freedom
DMARDs: Disease-modifying anti-rheumatic drugs
ESR: Erythrocyte sedimentation rate
GEE: Generalized estimating equation
g: Gram
mcg: Microgram
mg: Milligram
mHAQ: Modified Health Assessment Questionnaire
NSAIDs: Non-steroidal anti-inflammatory drugs
PGA: Patient global assessment
RCT: Randomized controlled trial
RA: Rheumatoid arthritis
RAH: Royal Adelaide Hospital
RAID: Rheumatoid Arthritis Impact of Disease
s.d.: Standard deviation
SF-36: 36-Item Short Form Survey
SHS: Sharp/van der Heijde
SA: South Australian
SJCs: Swollen joint counts
TJCs: Tender joint counts
T2T: Treat-to-target
VALI-RAI: Validated 5-item Rheumatology Attitudes Index
VAS: Visual analogue scale
VAS-GH: Visual analogue scale (VAS) of patient-reported disease activity
